# Beta-Actin Deficiency with Oxidative Posttranslational Modifications in Rett Syndrome Erythrocytes: Insights into an Altered Cytoskeletal Organization

**DOI:** 10.1371/journal.pone.0093181

**Published:** 2014-03-26

**Authors:** Alessio Cortelazzo, Claudio De Felice, Alessandra Pecorelli, Giuseppe Belmonte, Cinzia Signorini, Silvia Leoncini, Gloria Zollo, Antonietta Capone, Cinzia Della Giovampaola, Claudia Sticozzi, Giuseppe Valacchi, Lucia Ciccoli, Roberto Guerranti, Joussef Hayek

**Affiliations:** 1 Department of Medical Biotechnologies, University of Siena, Siena, Italy; 2 Child Neuropsychiatry Unit, University Hospital, Azienda Ospedaliera Universitaria Senese (AOUS), Siena, Italy; 3 Neonatal Intensive Care Unit, University Hospital, AOUS, Siena, Italy; 4 Department of Molecular and Developmental Medicine, University of Siena, Siena, Italy; 5 Department of Medical Sciences Surgical and Neuroscience, University Hospital, AOUS, Siena, Italy; 6 Department of Life Science, University of Siena, Siena, Italy; 7 Department of Sciences of Life and Biotechnologies, University of Ferrara, Ferrara, Italy; 8 Department of Food and Nutrition, Kyung Hee University, Seoul, South Korea; Institute of Genetics and Biophysics, Italy

## Abstract

Beta-actin, a critical player in cellular functions ranging from cell motility and the maintenance of cell shape to transcription regulation, was evaluated in the erythrocyte membranes from patients with typical Rett syndrome (RTT) and methyl CpG binding protein 2 (*MECP2*) gene mutations. RTT, affecting almost exclusively females with an average frequency of 1∶10,000 female live births, is considered the second commonest cause of severe cognitive impairment in the female gender. Evaluation of beta-actin was carried out in a comparative cohort study on red blood cells (RBCs), drawn from healthy control subjects and RTT patients using mass spectrometry-based quantitative analysis. We observed a decreased expression of the beta-actin isoforms (relative fold changes for spots 1, 2 and 3: −1.82±0.15, −2.15±0.06, and −2.59±0.48, respectively) in pathological RBCs. The results were validated by western blotting and immunofluorescence microscopy. In addition, beta-actin from RTT patients also showed a dramatic increase in oxidative posttranslational modifications (PTMs) as the result of its binding with the lipid peroxidation product 4-hydroxy-2-nonenal (4-HNE). Our findings demonstrate, for the first time, a beta-actin down-regulation and oxidative PTMs for RBCs of RTT patients, thus indicating an altered cytoskeletal organization.

## Introduction

Actins are highly conserved proteins ubiquitously expressed in eukaryotic cells [1]. In vertebrates, three main groups of actin isoforms have been identified, i.e., alpha, beta and gamma. While the alpha actins are found in muscle tissues and are a major constituent of the contractile apparatus, beta and gamma actins coexist in most cell types either as components of the cytoskeleton or mediators of internal cell motility [2]. In particular, beta-actin is a critical player in several cellular functions, ranging from cell motility and the maintenance of cell shape and polarity to transcription regulation. Interestingly, several syndromes associated with mental retardation are characterized by cortical dendritic abnormalities [3], and the neuronal cytoskeleton is essential for dendritic developmental processes. It includes microfilaments, neurofilaments and microtubules, each one being formed by a major protein, i.e., actin, neurofilament and tubulin, respectively [4], [5].

Rett syndrome (RTT), the second commonest cause of severe mental retardation in the female gender, is represented by a devastating neurodevelopmental disorder with a wide phenotypical heterogeneity which is caused in the overwhelming majority of cases by loss-of-function *de novo* sporadic mutations in the X-linked gene encoding the methyl-CpG binding protein 2 (MeCP2) [6]. In its classical phenotype, RTT is characterized by an apparently normal development in the first few months followed by loss of neurodevelopmental milestones in a 4-stage progression [7], [8]. Increasing evidence supports the concept that RTT is associated with impairment of dendritic arborization [9], [10]. Several studies have described neuronal abnormalities in RTT human brains, and in male Mecp2-mutant mice [9], [10], [11], [12], [13]. Overall, this body of research suggests that *MECP2* deficiency mutations are involved in cellular mechanisms regulating short term dynamics of dendritic spines early in development within the frame of maximum neural plasticity. However, the underlying cause for the dendritic arborization impairment in RTT is still largely unknown.

While prior studies on Mecp2-deficient brains show striking changes in neuronal maturation [14], recent evidence indicates that MeCP2 deficiency affects microtubule dynamics in RTT astrocytes and impairs microtubule stability in RTT primary fibroblast cultures [15], [16]. These data suggest that MeCP2 has a stabilizing role on microtubule dynamics and that its deficiency could lead to impaired microtubule stability which may at least partly underlie the dendritic abnormalities detected in RTT brains. We have previously demonstrated the existence of an abnormal erythrocyte shape in typical RTT patients showing a striking prevalence of circulating leptocytes with enhanced membrane oxidative stress (OS). Therefore, in the present study, we tested the hypothesis that a beta-actin deficiency with an increased oxidative posttranslational modification (PTMs) could underlie the red blood cells (RBCs) shape abnormalities in patients affected by the typical form of the disease [17].

## Materials and Methods

### Study Population and Ethical Statement

The study included 20 female patients with clinical diagnosis of typical RTT (median age: 5.0 years inter-quartile range 3–6, values range 3–10 years) with demonstrated *MECP2* gene mutations. RTT diagnosis and inclusion/exclusion criteria were based on RTT nomenclature consensus [18]. All the patients were admitted to the Siena Rett Syndrome National Reference Centre. Gender-matched healthy control subjects of comparable age (N = 20, median age: 5.0 years inter-quartile range 3–5.5, values range 3–10 years) with a typical development were selected as a control population. Blood samplings in the patients’ group was performed during the routine follow-up study at hospital admission, while the samples from the control group were carried out during routine health checks, sports, or blood donations, obtained during the periodic clinical checks. The subjects examined in this study were on a typical Mediterranean diet. The study was conducted with the approval by the competent Ethics Committee of the Azienda Ospedaliera Universitaria Senese, Siena. All the informed consents were obtained from either the parents or the legal tutors of the enrolled patients. Parents or legal tutors provided written informed consent to participate to the study, as approved by the Institutional Ethics Committee.

### Blood Sample Collection and Preparation

All samplings from RTT patients and healthy controls were carried out around 8 AM after overnight fasting. Blood was collected in heparinized tubes and all manipulations were carried out within 2 h after sample collection. The blood samples were centrifuged at 2400×g for 15 min at 4°C; after plasma and buffy coat removal, RBCs were washed twice with physiological solution (150 mM NaCl) and lysed in Dodge buffer containing 5 mM of potassium phosphate buffer, 0.5 mM EDTA, pH 8 and 1 mM of phenylmethanesulfonyl fluoride (Sigma). Erythrocyte membranes were freshly prepared, according to Dodge [19], by repeated washing until the “ghosts” were pearly white. Samples were kept frozen at −70°C until used. An aliquot of each blood sample (1 ml) was centrifuged at 800×g for 10 min at 4°C and washed twice with physiological solution for confocal microscopy analysis of erythrocytes.

### Immunoprecipitation of Erythrocyte Beta-actin

Ghosts (200 μg of protein determined using the protein assay; BioRad, Hercules, CA) were incubated with 5 μg of rabbit monoclonal anti-beta-actin antibody (cod. 04-1116; Millipore Corporation, Billerica, MA, USA) overnight at 4°C on a rotator. Then, immune complex was incubated with 50 μl of Protein A-Sepharose (Sigma-Aldrich, Milan, Italy) and rotated at 4°C for 2 h. Samples were centrifuged at 10,000×g for 5 min and washed three times with 1 ml ice-cold PBS. The pellet was mixed with 2 × reducing sample buffer, boiled and loaded on electrophoretic gels for silver staining or western blotting (WB) analysis. Samples processed equally but with normal rabbit IgG (Sigma-Aldrich, Milan, Italy), instead of anti-beta-actin antibody, were used as negative control.

### Polyacrylamide Gel Electrophoresis Analysis

Ghosts (20 μg of protein) and immunoprecipitated beta-actin were separated by sodium dodecyl sulfate polyacrylamide gel electrophoresis (10%, SDS-PAGE) according to Laemmli [20]. Resulting gels were stained with silver nitrate (Sigma-Aldrich, Milan, Italy). Gel images were acquired using LabScan software (GE Healthcare, version 6.0) by Image Scanner and the bands were automatically analyzed using TotalLab software (nonlinear dynamics, version 1.0). The application of the software allows calibration and normalization of the gel.

### Western Blot Analysis

Ghosts (40 μg of protein) and immunoprecipitated beta-actin were resolved on 10% SDS-PAGE gels and transferred onto nitrocellulose membranes (GE Healthcare Europe GmbH, Milan, Italy).

After blocking in 3% non-fat milk (BioRad, Hercules, CA, USA), the membranes were incubated overnight at 4°C with four antibodies (Abs) which recognize different beta-actin peptides: mouse beta-actin (anti-full length protein, amino acid (AA) sequence 1–375, cod. AB54724; Abcam, Cambridge, UK), mouse beta-actin (anti-N-terminal peptide, AA sequence 1–16, cod. A1978; Sigma-Aldrich, Milan, Italy), mouse beta-actin (anti-C-terminal peptide, AA sequence 364–375, cod. A3853; Sigma-Aldrich, Milan, Italy) and rabbit beta-actin (anti-C-terminal peptide, AA sequence not specified, cod. 04-1116; Millipore Corporation, Billerica, MA, USA). The beta-actin western blot analyses were performed using the four anti-beta-actin antibodies in comparison to the expression of the loading control GAPDH (anti-GAPDH, ab9484; Abcam, Cambridge, MA). Immunochemical detections of 4-HNE adducts on immunoprecipitated beta-actin and 2-DE RBC ghosts were performed using goat 4-HNE antibody (cod. AB5605; Millipore Corporation, Billerica, MA, USA).

Following washed in TBS Tween, membranes were incubated with specific secondary antibodies for 1 h at RT and, finally, with ECL reagent (BioRad, Hercules, CA, USA). Images were digitized (ChemiDoc XRS, BioRad, Hercules, CA). Optical densities were quantified using a computerized imaging system (Quantity One Imaging system).

### Confocal Microscopy

Erythrocytes purified as described before were smeared onto slides and after drying were fixed with 4% paraformaldehyde in phosphate buffered saline (PBS) at RT for 10 min, permeabilized with 0.1% Triton X-100 in PBS for 5 min at 4°C and washed three times in PBS. Cells were blocked with 1% BSA for 30 min at RT and incubated overnight at 4°C with a monoclonal rabbit beta-actin antibody (Millipore Corporation, Billerica, MA, USA) diluted 1∶50 in PBS. Following washed in TBS, cells were incubated with specific secondary antibody goat anti-rabbit (cod. A-11008; Alexa Fluor 488,) diluted 1∶100 in PBS for 1 h at RT. After three washes in PBS, fluorescent RBCs cells, mounted with a drop antifade, were visualized by confocal microscopy LSM-700 (Zeiss, Jena, Germany). Cell signal semiquantitative analysis and cells 3D visualization were carried out with ImageJ an open source program (rsb.info.nih.gov/ij).

### Two-Dimensional Gel Electrophoresis Analysis

Two-Dimensional gel electrophoresis (2-DE) was used to separate ghost proteins as previous described [21]. Samples (60 μg of protein determined using the Bradford method) [22], were denatured a solution containing 10% of SDS, 2.3% of dithiothreitol (DTT) heated to 95°C for 5 min. The samples were then combined with solubilizing buffer containing 8 M urea, 2% of 3-[(3-cholamidopropyl)-dimethylammonio]-1-propane sulfonate (CHAPS), 0.3% DTT, 2% immobilized pH gradient (IPG) buffer, and a trace of bromophenol blue and loaded into 18 cm IPG strips 3–10 non linear on an Ettan IPGphor (GE Healthcare) apparatus system and rehydrated for 7 h. Isoelectric focusing (IEF) was carried out for a total of 32 kV h. The strips were first equilibrated with a buffer containing 50 mM Tris-HCl, pH 8.8, 6 M urea, 2% w/v SDS, 30% v/v glycerol, and 1% w/v DTT for 15 min; then they were equilibrated again with the same buffer described above, except it contained 4% w/v iodoacetamide instead of DTT. The second dimension was performed on an EttanDalt Six Electrophoresis system (GE Healthcare). IPG strips were embedded at the top of a 1.5 mm thick vertical polyacrylamide gradient gel (8–16%T) using 0.5% w/v agarose and run at a constant current of 40 mA/gel at 20°C. Each sample was carried out in triplicate under the same conditions. For 2-DE/Western blot analysis, gels (containing 80 μg of protein) were transferred onto a nitrocellulose membrane (0.8 mA/cm^2^; 1 h, 40 min) with a Pharmacia Biotech Nova Blot semi-dry transfer instrument.

### Tryptic Digestion and Proteins Identification by Mass Spectrometry

A spot-picking list was generated and exported to Ettan Spot Picker (GE Healthcare). The spots were excised and delivered into 96-well microplates where they were destained and dehydrated with acetonitrile (ACN) for subsequent rehydration with trypsin solution. Tryptic digestion was carried out overnight at 37°C. Each protein spot digest (0.75 ml) was spotted into the MALDI instrument target and allowed to dry. Then 0.75 ml of the instrument matrix solution (saturated solution of α-cyano-4-hydroxycinnamic acid in 50% ACN and 0.5% v/v trifluoroacetic acid) was applied to dried samples and dried again. Mass spectra were obtained, as described [23], using an ultrafleXtreme MALDI-ToF/ToF (Bruker Corporation, Billerica, MA, United States). After tryptic peptide mass acquisition, mass fingerprint searching was carried out in Swiss-Prot/TREMBL and NCBInr databases using MASCOT (Matrix Science, London, UK, www.matrixscience.com).

### Image and Statistical Data Analysis

2-DE gel images were analyzed using ImageMaster 2D Platinum v7.0 software (GE Healthcare). Spot intensity was expressed as protein percentage volume (%V). Comparative analysis between healthy controls and RTT patients of differently expressed proteins were evaluated using either Mann-Whitney rank sum test or Kruskal-Wallis test. Statistical significance was indicated by a two-tailed *P*-value <0.05. Data were expressed as mean ± standard deviations (mean ± SD) from triplicate determinations obtained in five separate experiments. The MedCalc version 12.1.4 statistical software package (MedCalc Software, Mariakerke, Belgium) was used.

## Results

### Erythrocytes from RTT Patients Show a Decreased Beta-actin Band Intensity

**Figure 1:**
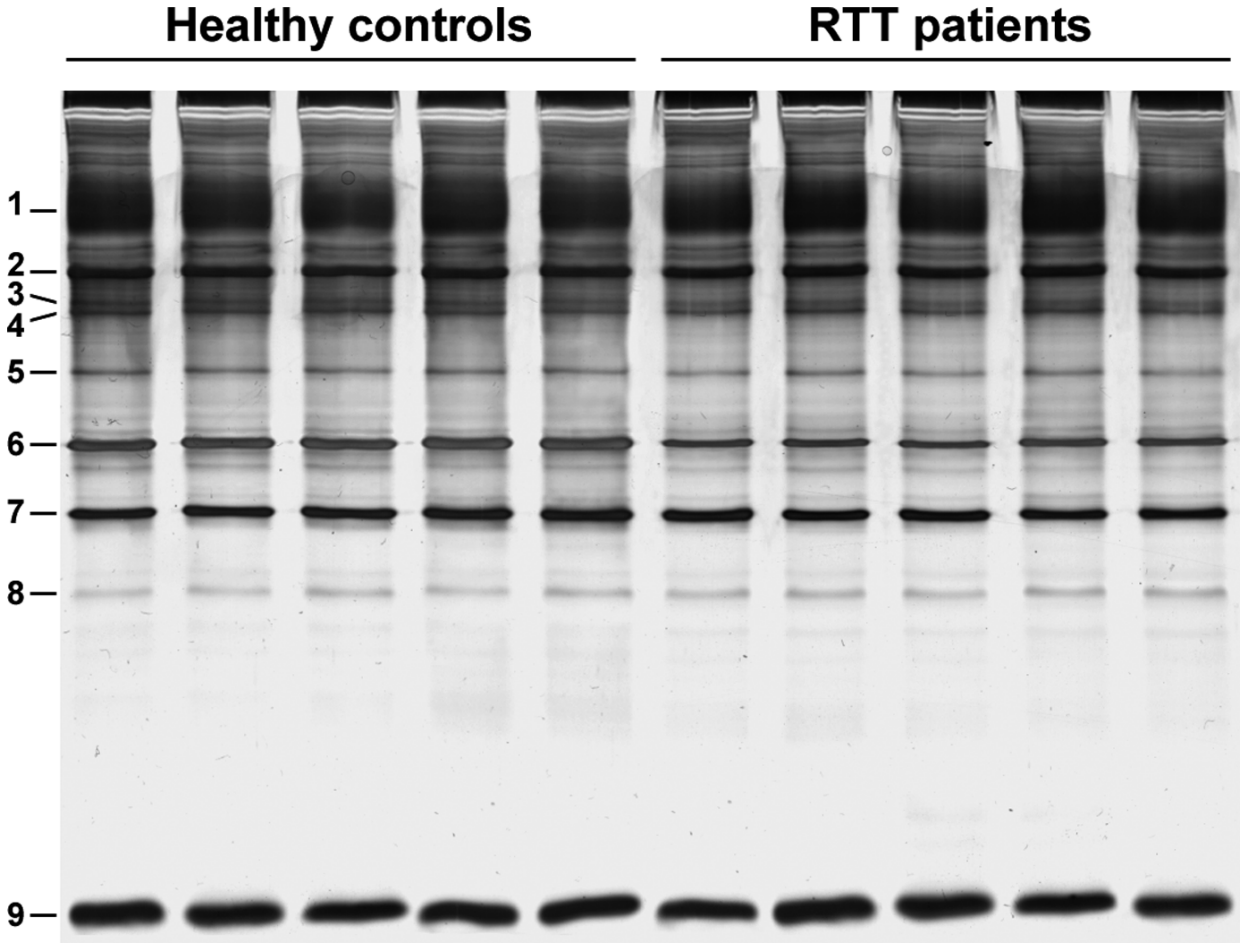
Beta-actin expression in RBC membranes. SDS-PAGE comparative analysis of RBC ghosts from healthy control subjects and RTT patients. Visible reduction of intensity for beta-actin (band 6) is present in RTT patients.

In the SDS-PAGE analysis (silver staining), a visible significant reduction (*P*<0.01) of the beta-actin (band 6) intensity was detected in the erythrocyte ghosts from RTT patients with an average decrease of −1.93±0.09 fold as mean ± SD (range: −2.09 to −1.78 fold), as compared to control values (Figure 1 and Table 1).

**Table 1:** Erythrocyte membrane beta-actin band decrease intensities in RTT patients.

Bandno.^(a)^	Healthy controls						RTT patients					
	Lane 1	Lane 2	Lane 3	Lane 4	Lane 5	Mean±SD	Lane 6	Lane 7	Lane 8	Lane 9	Lane 10	Mean±SD
**1**	31.11	29.56	29.35	31.06	29.99	30.21±0.82	29.02	29.04	29.31	31.67	30.60	29.92±1.17
**2**	10.60	10.85	10.31	10.91	10.87	10.70±0.25	10.44	10.78	10.36	10.91	10.57	10.61±0.23
**3**	2.72	2.70	2.57	2.51	2.46	2.59±0.11	2.67	2.71	2.53	2.58	2.39	2.57±0.12
**4**	2.89	2.78	2.66	2.58	2.52	2.68±0.14	2.59	2.79	2.42	2.48	2.40	2.53±0.16
**5**	2.76	2.78	2.65	2.6	2.58	2.67±0.09	2.51	2.74	2.71	2.80	2.5	2.65±0.13
**6**	**8.39**	**8.27**	**8.16**	**8.21**	**8.54**	**8.31±0.15**	**4.21**	**4.69**	**4.19**	**4.75**	**4.33**	**4.43±0.26** **
**7**	9.51	9.33	9.4	9.68	9.69	9.52±0.16	7.51	7.75	7.46	7.76	7.4	8.85±0.28
**8**	1.38	1.39	1.78	1.66	1.84	1.61±0.21	1.67	1.74	1.6	1.77	1.68	1.69±0.06
**9**	25.83	25.19	25.06	25.22	25.47	25.35±0.30	25.29	25.8	25.30	25.16	25.26	25.36±0.25

Result indicate band intensities as expressed as relative %V normalized values by SDS-PAGE analysis. Data are mean±SD.

a Band numbers refer to those of Figure 1. Bold characters indicate the beta-actin band intensity.

** = *P*<0.01.

### Beta-actin Decrease in RTT Erythrocytes is Independent from the Monoclonal Antibodies

**Figure 2:**
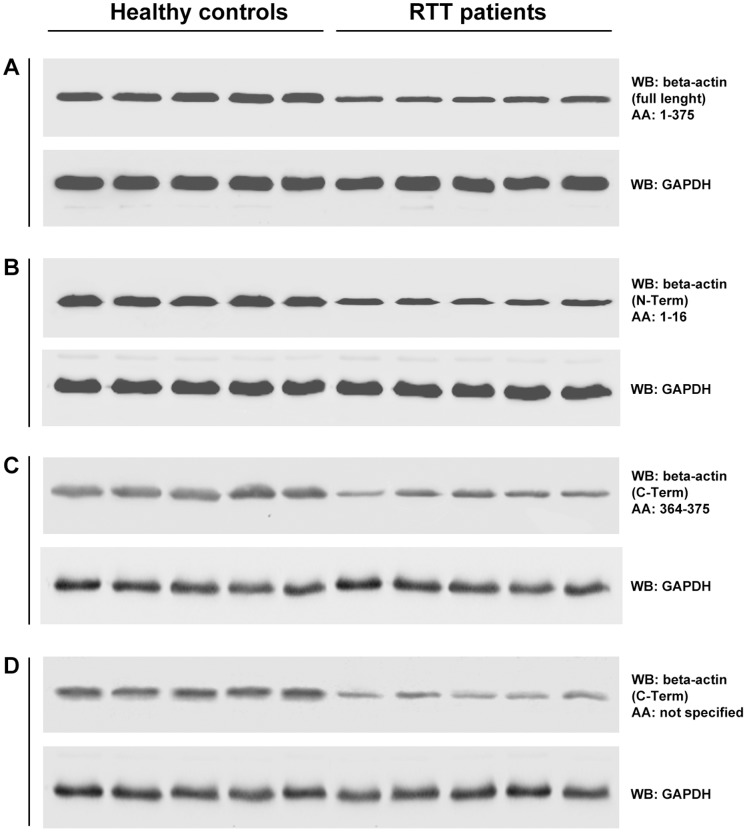
Western blot of beta-actin. Comparative analysis of RBC ghosts from healthy controls and RTT patients using four antibodies, which recognize different beta-actin amino acidic sequences (A: full length, B: N-terminal, C: C-terminal and D: C-terminal, amino acidic sequence not specified), confirm a beta-actin decrease in RTT patients.

Western blot analyses confirmed that the detected changes were specifically attributable to beta-actin, with a signal intensity decrease in the patients as compared to controls (Figure 2). Observed reduction in beta-actin band intensity is independent of the employed monoclonal antibodies (Figure 2A–D). These findings are compatible with a true decrease in beta-actin expression in the RTT patients erythrocytes membrane.

### RTT Erythrocytes Show Underexpressed Beta-actin Isoforms

**Figure 3:**
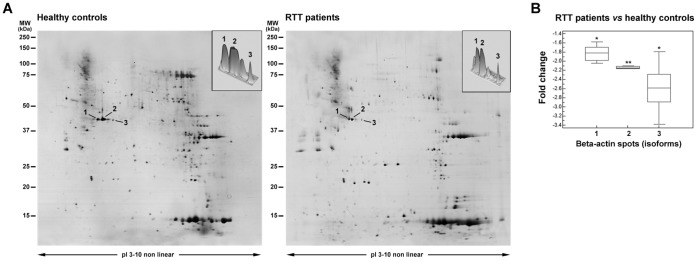
2-DE analysis of beta-actin isoforms. (A) Comparative analysis between RTT patients (right panel) and healthy controls (left panel). Arrows indicate the identified beta-actin isoforms which appear to be decrease in RTT patients. The top right panels represent the beta-actin differentially expressed spots on 3D view. (B) Quantitative analysis of the identified beta-actin spots changes in RTT patients as compared to control expression levels. Molecular mass and pI markers are indicated along the gels.

The 2-DE/MALDI-TOF analysis of RBC ghosts evidenced three differentially expressed beta-actin peptide spots both in RTT patients (Figure 3A, right panel) and in healthy controls (Figure 3A, left panel). A decrease in protein expression was detectable in each of the beta-actin RTT RBCs spots, as identified on the basis of their MW and pI values. Beta-actin spots identified by mass spectrometry, as well as peptide matches, sequence coverage, and the probabilistic score obtained using the MASCOT software are reported in Table 2. Quantitative analysis of the identified beta-actin spots changes (spot 1: −1.82±0.15 fold; spot 2: −2.15±0.06 fold; spot 3: −2.59±0.48 fold) are reported (Figure 3B).

**Table 2:** Identified beta-actin isoforms by 2-DE/MALDI-TOF analysis.

Spot no.^(a)^	AC^(b)^	p*I*/M_r_ (kDa)	p*I*/M_r_ (kDa)	Peptides	Sequence	MOWSE	Mean±SD	
		predicted	experimental	matches	coverage (%)	score	Healthy controls	RTT patients
**1**	P60709	5.14/42.1	5.15/42.0	9/17	23	156	1.51±0.13	0.98±0.09*
**2**	P60709	5.20/42.0	5.21/42.0	13/24	43	203	2.49±0.16	1.22±0.02**
**3**	P60709	5.27/42.0	5.29/41.9	9/16	17	115	0.18±0.04	0.08±0.01*

Result indicate spot intensities as expressed as relative %V normalized values. Data are mean±SD.

a Spot numbers are the same as shown in Figure 3.

b Accession numbers of Swiss-Prot or GenBanK databases.

* = *P*<0.05 or ** = *P*<0.01.

### Differential Expression and Distribution of Beta-actin in RTT Erythrocytes

**Figure 4:**
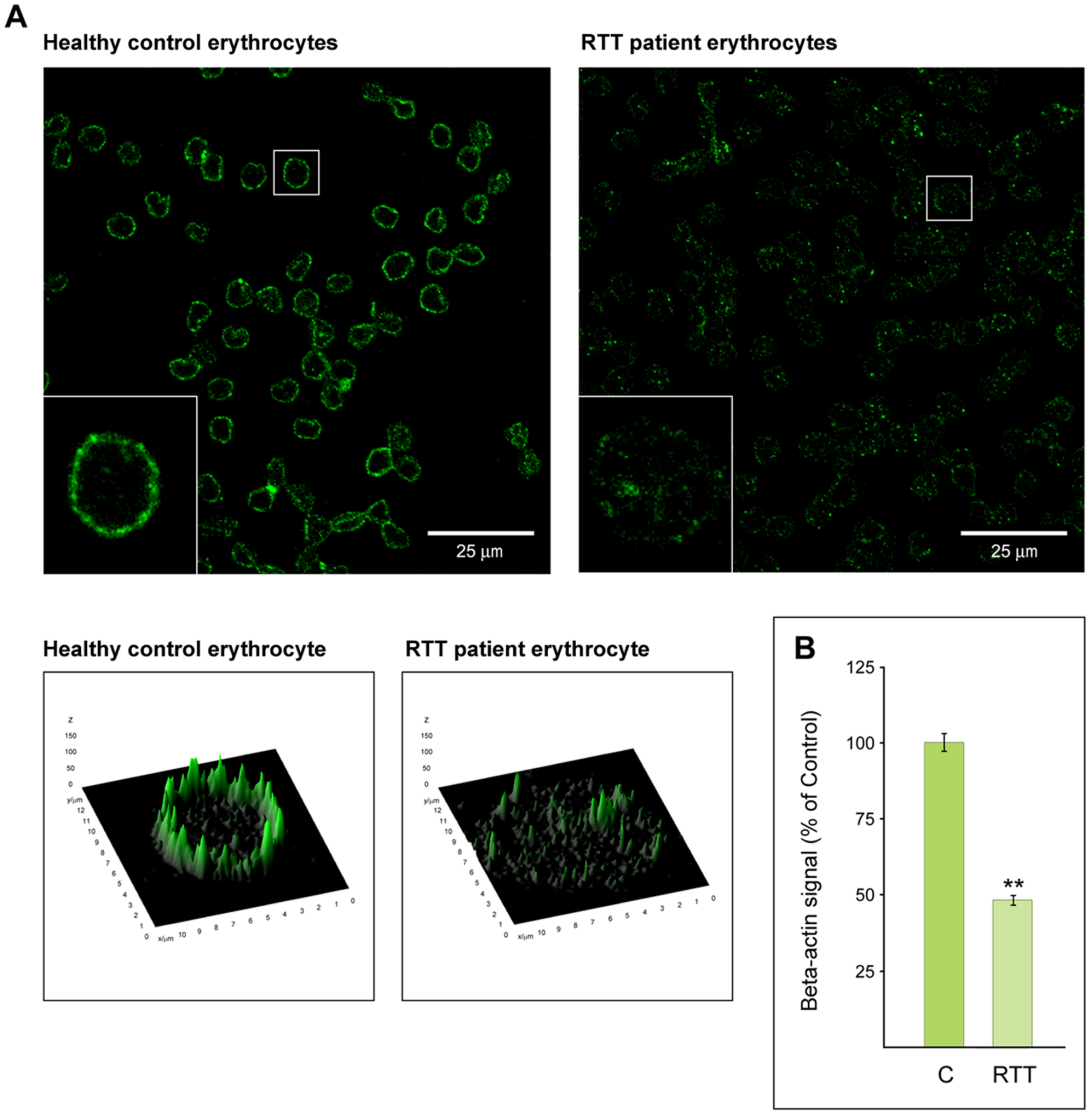
Confocal microscopy analysis of beta-actin distribution in RBCs. (A) Comparative analysis shows beta-actin expression and distribution differences between RBCs from RTT patients (right panel) and healthy control subjects (left panel) at confocal microscopy. Small panels represent, with flat and 3D views, two typical erythrocytes in which beta-actin signal is differentially distributed. Scale bar 25 μm. (B) Quantitative analysis of beta-actin signal differences in RBCs from healthy control (C) and RTT patients (RTT).

In the confocal microscopy analysis, beta-actin appears to be decreased and differently distributed in the RTT erythrocyte membranes (Figure 4A, right panel) as compared to healthy control RBCs (Figure 4A, left panel). As shown in Figure 4B, quantitative analysis of fluorescence signal intensity confirmed the decrement of beta-actin in RTT erythrocytes. These results indicate the existence of an altered cytoskeletal organization.

### Increased 4-HNE-beta-actin Adducts in RTT RBC Membranes

**Figure 5:**
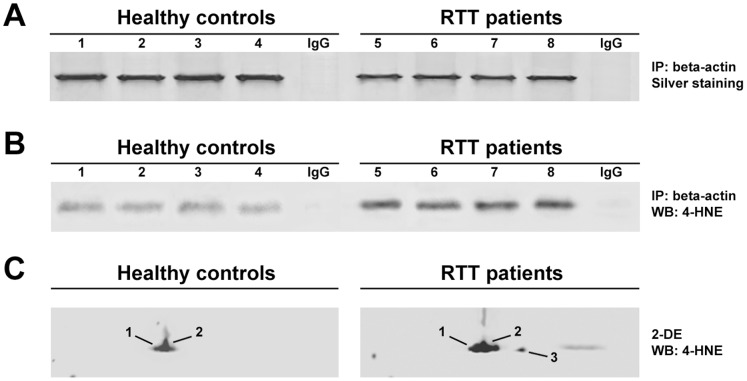
4-HNE/beta-actin adducts in RBC membranes. (A) SDS-PAGE (silver staining) of immunoprecipitated beta-actin from RBC ghosts of healthy control subjects (1–4) and RTT patients (5–8). (B) Western blot analysis of 4-HNE on immunoprecipitated beta-actin from RBC ghosts of healthy control subjects (1–4) and RTT patients (5–8). Immunoprecipitation (IP) with normal rabbit IgG served as a negative control. (C) 2-DE/Western blot analysis of 4-HNE on beta-actin from RBC ghosts proteome.

SDS-PAGE (silver stain) of immunoprecipitated beta-actin from RBC ghosts showed a visible reduction of intensity for the protein band in RTT patients (−1.84±0.05 fold; range: −1.9 to −1.7 fold) as compared to controls (Figure 5A). The absence of beta-actin in negative control IgG immunoprecipitates demonstrated that beta-actin was specifically precipitated from RBC ghosts. Immunochemical detection for 4-HNE adducts of immunoprecipitated beta-actin from RBC ghosts showed a significant increase in the signal intensity for aldehyde adducts in RTT patients (2.06±0.04 fold; range: 1.98 to 2.11 fold) as compared to controls (Figure 5B). 2-DE/Western blot for 4-HNE on RBC ghosts evidenced a significant increase in the signal intensity for aldehyde adducts in the three differentially expressed beta-actin peptide spots (Figure 5C).

## Discussion

The data of the present study demonstrate, for the first time, the occurrence of a beta-actin deficiency and its oxidative PTMs in the inner interface of the erythrocytes membrane from patients affected by RTT. The combination of these adverse factors can lead to an altered erythrocyte cytoskeletal network organization. Therefore, it is likely that a cytoskeletal disorganization is ubiquitously present in the cells of RTT patients. It is well known that beta-actin is an essential protein in the RBCs cytoskeleton which represents a major structural component for the horizontal tensile forces in the inner membrane [24]. The resulting imbalance between horizontal and vertical tensile forces could lead to the abnormal RBCs shape detectable in RTT.

On the other hand, a systemic OS is well established in RTT [17], [25], [26], [27], [28], [29], [30]. 4-HNE, formed by arachidonic acid or other unsaturated fatty acids following free radicals attack, can bind to proteins by Michael addition, with preferential amino acidic binding sites to cysteine (C), hystidine, (H), or lysine (K) residues [31]. Therefore, 4-HNE protein adducts could be considered a reliable OS markers and have a biological impact on protein function.

**Figure 6:**
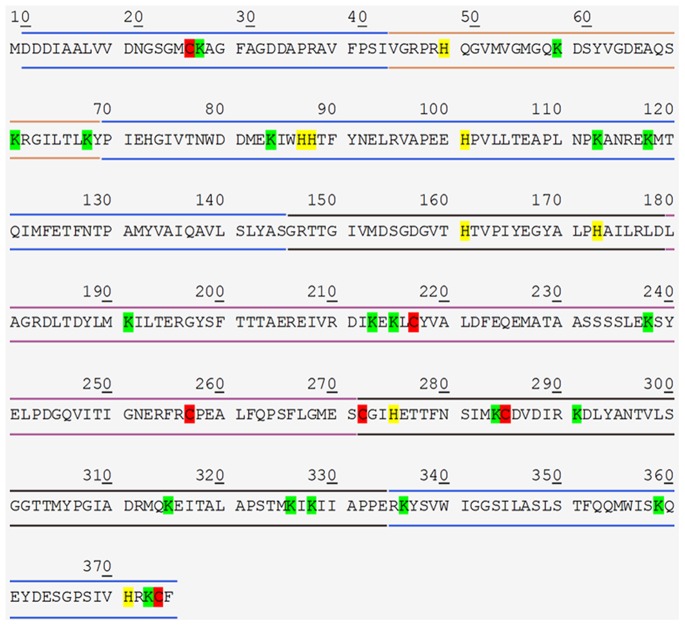
Potential binding sites for 4-HNE in the beta-actin amino acid sequence. The potential binding sites for 4-HNE are highlighted. Red color for 6 cysteine (C), yellow color for 8 hystidine (H) and green color for 19 lysine (K) residues. Blue double lines indicate sub-domain 1; orange lines indicate sub-domain 2; black lines indicate sub-domain 3 and purple lines indicate sub-domain 4 (primary sequence extracted from the ExPASy: SIB Bioinformatics Resource Portal, http://www.expasy.org/).

In this regard, the amino acid sequence of the human beta-actin shows several potential 4-HNE binding sites on all the major conformational subdomains of the protein (Figure 6). A total of 6 C, 8 H and 19 K residues are present in the sequence, and potential 4-HNE binding amino acid residues are present in all the relevant conformational subdomains of the protein, for a total of 2 C, 4 H and 7 K in the subdomain 1, a total of 1 H and 3 K in the subdomain 2, a total of 2 C, 2 H and 5 K in the subdomain 3, and a total of 3 C and 4 K in the subdomain 4 [32]. In particular, the subdomain 2 of the molecule is of critical importance for DNAase 1 binding and protein polymerization, while a very crucial C 374 residue and potential binding site for 4-HNE is present in the conformationally critical hydrophobic pocket of the molecule [32].

Our findings indicate that the observed reduction in beta-actin band intensity is independent of the employed monoclonal antibodies and are compatible with a real decrease in the beta-actin expression in the RTT patients erythrocytes membrane. Moreover, the reported evidence exhaustively addresses the question of whether the observed decrease in beta-actin protein expression might be related to oxidative PTMs potentially preventing antibody recognition. Taken together, our data indicate that major alterations exist in the beta-actin of RTT erythrocytes which results both from changes in expression and as a consequence of oxidative damage. To this regard, beta-actin is a well-known major target for OS processes [33], while erythrocytes represent “cellular detectors” revealing the co-existence of major cytoskeletal changes in this key genetic model for neurodevelopmental disorders. In particular, this work suggests a novel role for MeCP2 as a stabilizing protein in microtubule dynamics.

To date, no definitive cure for RTT exists, although several approaches to a potential therapy have been either attempted or hypothesized, including activation of the silenced *Mecp2* gene [34], [35], gene therapy [36], modulation of some of the downstream effects from MeCP2-deficiency [37]. Our study indicates that a proteomic approach in RTT is able to reveal additional downstream effects of the *MECP2*-deficiency, and therefore could identify potentially novel therapeutical targets for the disease.
